# Prenatal Factors in the Development of Allergic Diseases

**DOI:** 10.3390/ijms25126359

**Published:** 2024-06-08

**Authors:** Manuela Grijincu, Maria-Roxana Buzan, Lauriana-Eunice Zbîrcea, Virgil Păunescu, Carmen Panaitescu

**Affiliations:** 1 Center of Immuno-Physiology and Biotechnologies, Department of Functional Sciences, Victor Babeș University of Medicine and Pharmacy, 300041 Timișoara, Romania; 2 OncoGen Center, Pius Brînzeu County Clinical Emergency Hospital, 300723 Timișoara, Romania

**Keywords:** allergy development, allergy progression, parental history, exposure, cord blood IgE

## Abstract

Allergic diseases are showing increasing prevalence in Western societies. They are characterized by a heightened reactivity towards otherwise harmless environmental stimuli. Allergic diseases showing a wide range of severity of symptoms have a significant impact on the quality of life of affected individuals. This study aims to highlight the mechanisms that induce these reactions, how they progress, and which prenatal factors influence their development. Most frequently, the reaction is mediated by immunoglobulin E (IgE) produced by B cells, which binds to the surface of mast cells and basophils and triggers an inflammatory response. The antibody response is triggered by a shift in T-cell immune response. The symptoms often start in early childhood with eczema or atopic dermatitis and progress to allergic asthma in adolescence. An important determinant of allergic diseases seems to be parental, especially maternal history of allergy. Around 30% of children of allergic mothers develop allergic sensitization in childhood. Genes involved in the regulation of the epithelial barrier function and the T-cell response were found to affect the predisposition to developing allergic disorders. Cord blood IgE was found to be a promising predictor of allergic disease development. Fetal B cells produce IgE starting at the 20th gestation week. These fetal B cells could be sensitized together with mast cells by maternal IgE and IgE–allergen complexes crossing the placental barrier via the low-affinity IgE receptor. Various factors were found to facilitate these sensitizations, including pesticides, drugs, exposure to cigarette smoke and maternal uncontrolled asthma. Prenatal exposure to microbial infections and maternal IgG appeared to play a role in the regulation of T-cell response, indicating a protective effect against allergy development. Additional preventive factors were dietary intake of vitamin D and omega 3 fatty acids as well as decreased maternal IgE levels. The effect of exposure to food allergens during pregnancy was inconclusive, with studies having found both sensitizing and protective effects. In conclusion, prenatal factors including genetics, epigenetics and fetal environmental factors have an important role in the development of allergic disorders in later life. Children with a genetic predisposition are at risk when exposed to cigarette smoke as well as increased maternal IgE in the prenatal period. Maternal diet during pregnancy and immunization against certain allergens could help in the prevention of allergy in predisposed children.

## 1. Introduction

Allergic diseases are an increasing health problem, with one in four people in the Western world predicted to become allergic [1,2]. The clinical manifestations of allergies show a wide range of severity from mild local symptoms to severe systemic reactions. The mild local symptoms include rhinitis, conjunctivitis and skin symptoms, whereas severe symptoms are asthma and anaphylaxis. Currently, 400 million people worldwide are diagnosed with allergic rhinitis, which is often accompanied by symptoms of conjunctivitis, and 300 million suffer from asthma [2,3]. Therefore, allergic diseases imply both direct costs for the patients in terms of the financial burden for the treatment of symptoms as well as indirect costs by affecting quality of life, work performance, productivity and social life [4]. Treatments of allergic disease include symptomatic treatment and allergen-specific immunotherapy, which aims to redirect the immune response towards a tolerogenic reaction [5]. These treatments can be both costly and, in some cases, ineffective, making the development of prevention strategies a priority. The identification of the risk and protective factors in the development of allergic diseases could provide an important step in the treatment of these disorders. Therefore, this study aims to review the mechanisms involved in allergic sensitization and the progression of allergic diseases in order to highlight which and how prenatal risk and protective factors are involved in the development of allergic diseases.

### 1.1. Mechanisms Inducing Allergy

Allergy is defined as an exaggerated immune response towards harmless environmental constituents [6]. The most frequent elicitors of allergic reactions are pollen, house dust mites (HDMs), food allergens, insect venom and drugs [7]. These exaggerated immune responses are known as hypersensitivity reactions. According to a position paper by the European Academy of Allergy and Clinical Immunology (EAACI), hypersensitivity reactions are now comprised of nine types of reactions: types I–III consist of antibody-mediated hypersensitivities, IVa–c are cell-mediated hypersensitivities, V–VI imply a tissue-mediated mechanism and type VII is the direct response to chemicals [8]. Atopy was classified as a type I hypersensitivity, characterized as the personal or familial tendency to produce immunoglobulin E (IgE) antibodies against environmental proteins, which upon cross-linking induce symptoms of asthma, rhino-conjunctivitis or dermatitis [6]. According to the new EAACI position paper, the term “atopy” should no longer be used due to limited relevance in describing the different disease phenotypes [8]. The type I hypersensitivity response is intertwined with the type IVb cellular hypersensitivity response, since the T cells induce the class switch in B cells to produce IgE [8]. The type IV immune response in driven by the differentiation of T cells. The IVa response is driven by type 1 T helper cells (Th1), which produce mainly interferon-gamma (IFN-γ), interleukin-2 (IL-2) and IL-12. On the other hand, in the type IVb response, type 2 T helper cells (Th2) secrete IL-4, IL-5, IL-10 and IL-13. These cytokines mediate the production of IgE and the activation of basophils, which are inducers of type I hypersensitivity [9] (Table 1). Failures in type I and type III cellular responses are causes for autoimmune diseases (rheumatoid arthritis, autoimmune gastritis and Hashimoto thyroiditis), whereas malfunctions in type II responses, i.e., an imbalance between Th1/Th2 skewed towards Th2, trigger allergic diseases [10].

**Figure 1:**
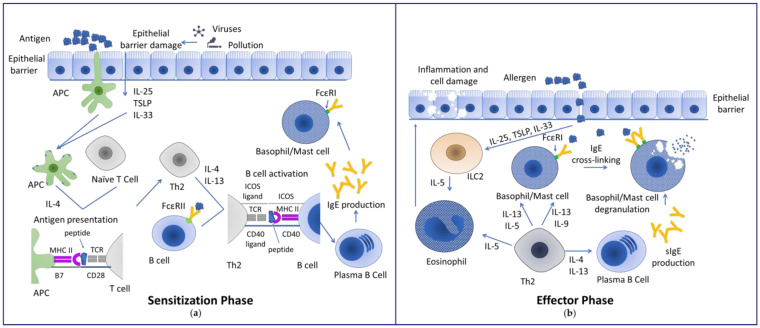
Representation of the mechanisms involved in the sensitization and effector phase of allergic response adapted from [12,17,18,19]: (**a**) During the sensitization phase, the antigen is taken up by APC either by APC screening or by APC recruitment upon alarmin secretion (IL-25, TSLP and IL-33) by epithelial cells and processed into peptides. The peptides are presented to naïve T cells via MHC II molecules. Under IL-4 secretion, the T cells develop into Th2. The IgE bound antigen is recognized by B cells via FcεRII and processed into peptides. The peptides are recognized by Th2 cells, which upon secretion of IL-4 and IL-13 induce class switching of B cells to IgE-producing plasma cells. The IgE produced by these cells binds to the FcεRI on the surface of mast cells and basophils; (**b**) During the effector phase, upon the second encounter, the antigen is now recognized as an allergen. Allergen recognition is able to induce cross-linking of the IgE receptors on the surface of basophils and mast cells, releasing inflammatory mediators (histamine, leukotrienes, etc.). These mediators then trigger the allergic symptoms. Th2 and ILC2 play an important role by secreting IL-5, thereby activating and recruiting eosinophils at the site of the allergen encounter, which can induce chronic inflammation upon degranulation. Additionally, by IL-4 and IL-33 secretion, Th2 cells stimulate the production of specific IgE in plasma B cells. APC—antigen presenting cells, FcεRI—high-affinity IgE receptor, FcεRII (CD23)—low-affinity IgE receptor, ICOS—inducible costimulatory antigen, IL—interleukin, ILC2—type 2 innate lymphoid cells, MHC II—class II major histocompatibility complex molecules, TCR—T-cell receptor, Th2—type 2 T helper cell, TSLP—thymic stromal lymphopoietin.

The allergic response is divided into two phases, an initial sensitization phase and an effector phase (Figure 1). During the sensitization phase, antigen presenting cells (APC) come into contact with an antigen (allergen) at the barrier (lung, skin and gut) and then incorporate the allergen. The allergen is divided into peptides, which are presented via the class II major histocompatibility complex molecules (MHC II) on the cell surface to naïve T cells. The APC can then drive the differentiation of T cells in the thymus by secreting different mediators. The type 2 differentiation into Th2 and type 2 cytotoxic T cells (Tc2) is driven by IL-4, secreted by mast cells and basophils. IL-4 secretion is sustained by type 2 innate lymphoid cell (ILC2) signaling under the stimulation of epithelial cell alarmins and cytokines in response to infections or pollution [8,11,12] (Figure 1a). IL-4 and IL-13 secreted by Th2 cells induce the class switching to IgE production in B cells and stimulate the tissue migration of T cells [8]. Mediators released by follicular T helper cells in response to allergens induce the production of allergen-specific high-affinity IgE [13].

During the effector phase, upon subsequent encounters with the antigen, mast cells and basophil expressing the high-affinity IgE receptor (FcεRI) on the cell surface become sensitized to the allergen by the binding of specific IgE (sIgE) to these receptors. Allergen encounter and recognition induces the cross-linking of sIgE on the cell surface and cell degranulation. Upon degranulation, inflammatory mediators like heparin, histamine and leukotrienes are released and cause the known allergic symptoms, from vasodilation and mucus production to anaphylaxis [14,15]. T cells and type 2 innate lymphoid cells (ILC2) further release IL-5 and IL-13, which activate eosinophils that can cause tissue damage upon degranulation, leading to chronic inflammation in asthma and food allergy in type IVb hypersensitivity reactions [8,16] (Figure 1b).

**Figure 2:**
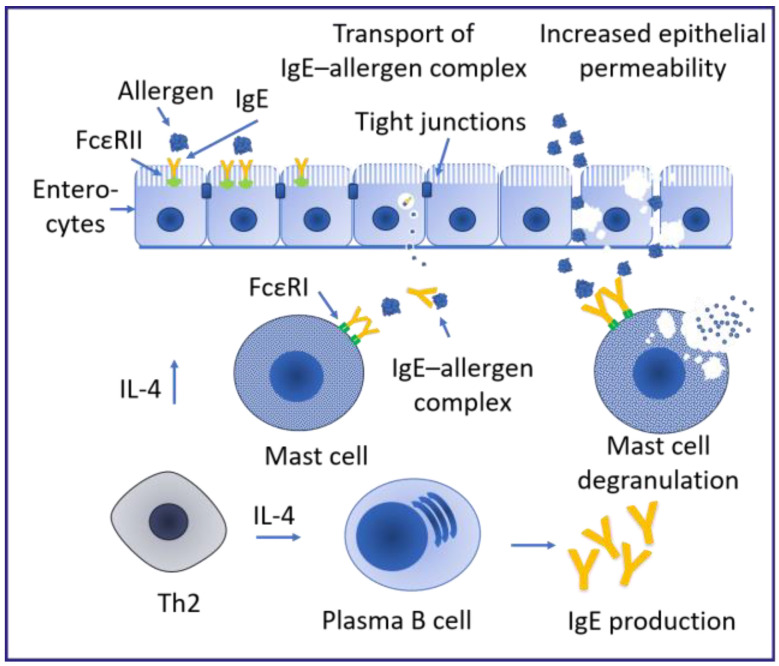
Role of CD23 (FcεRII) on enterocytes in regulating the inflammatory response in food allergy adapted from Ristivojević et al. [30]. IL-4 secreted by T cells increases the expression of the low-affinity IgE receptor (FcεRII, CD23) on the surface of enterocytes and induces the production of IgE in plasma B cells. Upon cross-linking of FcεRII on the surface of enterocytes, IgE and IgE–allergen complexes are taken up by the enterocytes and transported across the epithelial barrier without degradation. The IgE and allergen are exposed to the underlying immune system. The allergen can then bind to the high-affinity IgE receptor (FcεRI) on mast cells and induce degranulation. The degranulation of mast cells increases the membrane permeability and facilitates further allergen uptake. FcεRI—low-affinity IgE receptor (CD23), FcεRII—high-affinity IgE receptor, IgE—immunoglobulin E, IL-4—interleukin 4, Th2—type 2 T helper cell.

The FcεRI on basophils and mast cells is important for binding and cross-linking IgE to trigger the inflammatory response upon allergen contact. However, the low-affinity IgE receptor CD23 (FcεRII) was described as playing a role in the regulation of the allergic response [20,21] (Figure 2). CD23 oligomers on the surface of B cells showed better IgE binding if the antibody formed larger complexes with the antigen [22]. In particular, allergen-bound IgE showed a stronger affinity for the CD23 receptor [23]. Cross-linking was found to induce the internalization of the recognized allergen by either trans- or endocytosis [21,24]. The cross-linking of the CD23 receptor was found to induce different signaling cascades on B cells and monocytes, regulating the availability of free circulating IgE [25,26,27]. Additionally, the uptake of IgE–antigen complexes in B cells via CD23 resulted in a facilitated antigen presentation to T cells and an increase in antigen-specific T cells [28]. On enterocytes, CD23 expression was upregulated upon IL-4 stimulation, which enabled the IgE transport across the epithelial barrier [29,30] (Figure 2). Additionally, enterocytes expressing CD23 facilitated the transport of IgE–allergen complexes across the epithelial barrier in sensitized mice, thereby indicating a role of this receptor in food allergies [30,31] (Figure 2). The uptake of the IgE–allergen complex was found to upregulate IL-8 and chemokine ligand 20 (CCL20), triggering the migration of dendritic cells and potentially eosinophils in the area, thereby inducing inflammation [12,32]. The inflammation in addition to the degradation of tight junctions by the protease activity of certain food allergens further facilitates the increased transport of allergens across the barrier and leads to chronic inflammation [8,33] (Figure 1b).

### 1.2. Disease Progression in Association with Sensitizations

**Figure 3:**
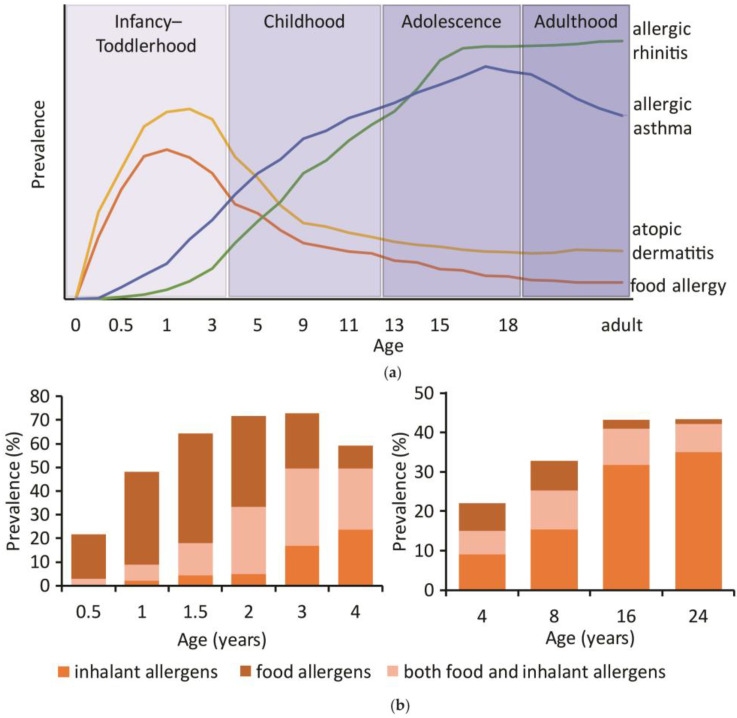
Progression of allergy and sensitization over time: (**a**) progression of allergic manifestations from infancy to adulthood adapted from Tsuge et al. [42]; (**b**) progression of sensitization towards food, inhalant allergens and both food and inhalant allergens in infancy and toddlerhood adapted after Chiu et al. [43] and from childhood to adulthood adapted from Melén et al. [44].

The clinical manifestations of allergic diseases in infancy and childhood are usually skin symptoms, such as eczema, rarely associated with rhinitis or asthma [34] (Figure 3a). The skin reactions are often in response to food allergens. As the children grow older, respiratory symptoms such as asthma and rhinitis tend to become more prevalent, sometimes accompanied by skin symptoms (Figure 3a). This progression of allergic disorders from skin symptoms in childhood to respiratory symptoms in adolescence is known as atopic march [35]. Several studies have found evidence for this progression from early atopic dermatitis (AD) and eczema to allergic rhinitis and asthma, while also considering the severity and persistence of symptoms [36,37] (Figure 3a). The symptom progression was often associated with an increase in total or specific IgE (sIgE) levels [36] (Figure 3b). Therefore, the inclusion of different disease phenotypes, comprising the onset, severity, persistence and co-occurrence of skin symptoms, respiratory symptoms and allergen sensitizations, has led to the identification of up to seven disease trajectories [38,39,40,41]. Important risk factors for the development of allergic diseases in adolescence have been identified, which include early dermatitis or bronchial infections and sensitization to certain risk molecules [38,41].

Sensitizations often occur in order of encounter with the allergens, which are accompanied by elevated IgE levels [45]. Early-life food sensitizers include hen’s eggs, cow’s milk, fish and peanuts [45,46,47]. Food sensitizations in toddlerhood were identified as risk factors for skin and respiratory symptoms in childhood [48,49], while skin symptoms in toddlerhood were risk factors for respiratory symptoms in adolescence and adulthood [50]. Sensitizations towards hen’s egg and milk decreased from toddlerhood to childhood, yet peanut sensitization appeared to remain until adulthood [43,44]. In late toddlerhood and childhood, sensitizations towards inhalant allergens start to develop, and the most frequent sensitizers include house dust mites (HDMs), animal dander and pollen [36,47] (Figure 3b). Sensitization to inhalant allergens was found to increase as the children aged, reaching a 42.2% prevalence in young adults, while food allergen sensitization decreased to 8.2% [44] (Figure 3b). These sensitizations become risk factors for the development of asthma in adolescence [48]. Early-life sensitization to certain risk molecules, e.g., Der p 23 from HDM, Fel d 1 from cat dander or Phl p 1 and Phl p 4 from timothy grass pollen, were predictive of respiratory allergy in adolescence and adulthood [39,51,52,53]. Allergic multimorbidity was associated with both food and inhalant allergen sensitization at different time points in life, with increasing sensitizations being risk factors for more persistent and severe symptoms [41,54]. Conversely, a lower level of sIgE and fewer additional inhalant allergen sensitizations in adolescence were indicative of the remission of allergic rhinitis between 16 and 24 years [50].

## 2. Prenatal Risk Factors in the Development of Allergic Diseases

The key components in the type II immune response are shifted differentiation towards Th2 cells as well as the production of IgE antibodies (Table 1). This imbalance in Th1/Th2 response is attributed to multiple factors, including wide use of antibiotics, early exposure to high allergen loads, increased allergenic potency due to pollution and fewer infectious contacts due to better hygiene and living conditions [9,55]. Therefore, investigations on the prenatal factors determining allergic diseases involve determining the prenatal presence of/the exposure to IgE antibodies and cytokines stimulating the Th2 response. Studies examining prenatal factors in the development of allergies have measured one or more of the following outcomes associated with these disorders: lung function (forced expiratory volume (FEV), fractional exhaled nitric oxide (FeNO), forced vital capacity (FVC), forced expiratory volume in one second (FEV1) and forced mid-expiratory flow (FEF_25–75%_)), allergen sensitization, atopic dermatitis, asthma, eczema, total IgE and specific IgE towards food or inhalant allergens.

**Figure 4:**
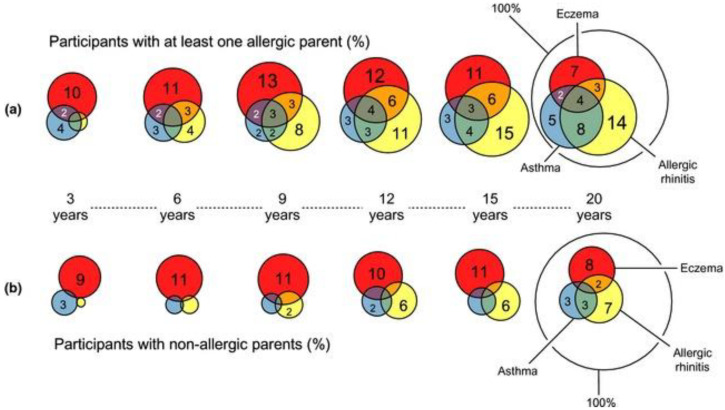
Prevalence of allergic multimorbidity over 20 years in patients with and without family history of allergic disorders after Gough et al. [34] (license no. 5778240499608): (**a**) prevalence of allergic diseases over time in patients with parental history of allergy; (**b**) prevalence of allergic diseases over time in patients without parental history of allergy. The colors represent the different allergic morbidities: allergic rhinitis (yellow), asthma (blue) and eczema (red), whereas their overlap indicates the occurrence of multimorbidity. The numbers represent the percentages for each morbidity for each age group.

Previous multigenerational studies have found that 64% of allergic individuals had a family history of this disorder, whereas only 31% of non-allergic individuals had a family history of allergy [56]. A cohort study performed in Germany found that over time, the prevalence of allergic diseases up to 20 years was higher in children with a family history [34]. This effect was more pronounced in males than in females [34]. In early childhood, the difference between children with and without a family history of allergy was not particularly pronounced, with the most common symptom being eczema (Figure 4). Starting with 6 years of age, children with a parental history of allergy started to develop allergic rhinitis, the proportion of patients being higher in males [34]. Additionally, with increasing age from 3 to 20 years, the percentage of patients lacking allergic manifestations decreased, the effect being stronger in individuals with a family history of allergy (Figure 4). Allergic multimorbidity in patients with a family history was more prevalent in males than in females [34]. The higher proportion of multimorbidity over time in males could be driven by more frequent IgE sensitization to inhalant allergens [44].

Maternal allergic disorders were significant risk factors for allergic diseases in early childhood, and maternal asthma was found to result in a threefold higher asthma risk in children [57,58]. Maternal asthma and allergy increased the risk for different allergic disorders in children, whereas postnatal smoking was a risk factor for recurrent wheezing in children [59,60,61]. Risk factors for the development of AD during the first 2 years of life were maternal and paternal history of AD, while wheezing bronchitis was mostly associated with a maternal history of asthma [46]. There is evidence for parental history playing an important role in the development of both inhalant and food allergies of offspring from infancy to childhood, with multiple food sensitizations being more frequent among children with a parental history of allergic rhinitis [48,62,63].

Maternal allergy could trigger the onset of allergies in offspring either by the inheritance of allergic predisposition and/or by creating an environment which could favor the development of allergic disorders. Several studies have found evidence supporting genetic predisposition. Twin studies have revealed that IgE levels have a genetic component, as the variation in IgE levels was found to be a lot smaller among twins than in the overall population. Additionally, the differences were smaller among monozygotic twins than among dizygotic twins. The variation in IgE levels was smaller among children than among adults, which could be indicative of environmental effects [64]. The investigation of food allergy among twin pairs also identified genetics as a risk factor in allergy development [65]. A recent twin study also showed that a genetic component drove both asthma and allergic sensitization in children [66].

**Figure 5:**
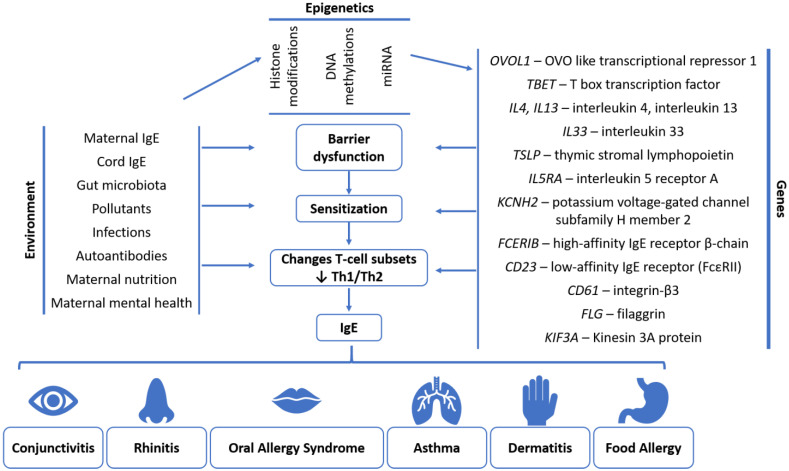
Environmental, epigenetic and genetic factors leading to the development of allergic diseases adapted after [37,71,75,83,84,85,86,87]. The main drivers of allergic diseases are the dysfunctions of the epithelial barrier, allergic sensitization and differentiation of T cells. Environmental factors either directly influence these drivers of allergy development or they induce changes in gene expression by epigenetic modifications. Genetic factors include certain allele variants of genes involved in T-cell differentiation, barrier function or sensitization, which increase the susceptibility to allergy development.

The locus 11q13 associated with allergic disorders was found to be maternally inherited [67]. Further genetic determinants of allergic diseases have been identified in genome-wide association studies (GWAS). Single-nucleotide polymorphisms (SNPs) in the MHC class II genes *HLA-DRB1*/*DQA1*/*DQB1* were found to be associated with the allergic sensitization towards certain inhalant allergens [68]. Similarly, seven out of nine SNPs associated with IgE were related to the development of allergic disorders [69] (Figure 5). Park et al. found that the heritability of SNPs and family history were predictors of asthma and AD in infants, while also highlighting an increasing importance of environmental factors as children age [70]. GWAS have also identified susceptibility genes associated with allergic rhinitis, asthma and atopic dermatitis [71]. These genes are involved in the regulation of the epithelial barrier function, as well as T-cell differentiation—T-cell function and regulation, Th2 immunity and transforming growth factor beta (TGF-β) signaling [71,72]. The loss of function of *FLG* filaggrin in the epithelium was associated with the risk of atopic dermatitis and atopic march due to dysfunction of the epithelial barrier [37,73]. Similarly, a risk allele in the *KIF3A* gene encoding the ciliary Kinesin 3A protein was found to be associated with the risk of allergen sensitization and asthma development [74] (Figure 5). These genes also play a role in the development of AD, with differences having been reported between AD patients and healthy subjects in the regulation of immune responses via a shift towards Th2 immunity [75].

Additionally, epigenetic effects that do not induce changes in the genetic code but modify the transcription of genes by DNA methylation, histone modification and microRNA (miRNA) have been investigated regarding their role in allergy development [75,76] (Figure 5). Maternal allergy was found to induce methylation patterns that were detected in cord blood [77]. A meta-analysis identified thirty-four CpG sites associated with asthma outcomes in infants, and seven CpG sites showed higher methylation in newborn blood samples of children who developed asthma at school age [78]. Among the CpG sites, those involved in regulating IL-5 receptor A *(IL5RA*) and potassium voltage-gated channel subfamily H member 2 (*KCNH2*) genes have been proposed as drug targets [78] (Figure 5). DNA/histone methylation and histone acetylation were proposed to drive the allergic inflammatory response by regulating the differentiation of T cells, i.e., via modifications in the promotor regions of *GATA3*, *RORC*, *PU1 IRF4*, *FOXP3* and *TBX1* transcription factors [75,79]. A recent review highlights the role of miRNAs in the development of allergic disease by showing how the expression of these miRNAs is influenced by environmental exposure to pollutants and allergens, further regulating genes involved in oxidative stress and T-cell differentiation [80]. Smoking and environmental toluene and benzene induced a higher expression of miRNA in maternal and cord blood, which was further correlated with a reduced number of regulatory T cells and an increased risk of AD in offspring [81]. Similarly, different DNA methylation patterns were associated with the duration and the window of exposure to cigarette smoke during pregnancy, with some of the identified methylation signatures persisting into adolescence [82].

In addition to genetic and epigenetic effects, the in utero environment could also contribute to the development of allergies in infants. Maternal allergy could influence allergy development by exposing the fetus to elevated levels of IgE through the umbilical cord, since maternal serum IgE levels were found to also increase cord blood IgE levels [88,89]. In a clinical study investigating 613 childbirths, 53% of infants had elevated cord blood IgE [90]. The main drivers of cord blood IgE were maternal allergy and pesticide use, whereas among the allergic mothers, the season of birth in spring was associated with elevated cord blood IgE [90]. The origin of cord blood IgE was not entirely identified, with studies having reported evidence for both fetal and maternal origin. Since fetal cells were shown to produce IgE starting with the 20th week of gestation and cord blood IgE levels did not show a proportional change to maternal serum IgE, some studies claimed a fetal origin of these antibodies [91,92]. However, a more recent study claimed that cord blood IgE was of maternal origin, since maternal- and fetal-specific antibodies showed strong correlations [93,94,95]. A total of 34.2% of children with elevated cord blood IgE (>0.5 kU/L) became sensitized to inhalant allergens by age 4 compared to only 18.1% with low/not detectable cord blood IgE levels [96]. Elevated cord blood IgE (>0.5 kU/L) resulted in an increased risk of eczema at age 4 [57], as well as a three times higher risk of allergic sensitization in infancy [57], but not for allergic asthma [58]. In addition to IgE, the infant could also come into contact with the allergen during the fetal development. Major food allergens from cow’s milk, hen’s eggs, peaches and wheat have been detected in amniotic fluid [97]. IgE antibodies have also been detected in amniotic fluid, showing strong correlation with maternal IgE levels [98]. Bertino et al. also measured sIgE against different cow’s milk proteins and found a strong correlation between maternal and fetal sIgE [94]. Some studies have found that peanut consumption during pregnancy increased the risk of infant peanut allergy, yet a systematic review did not reveal any effect of allergen avoidance during pregnancy on infant allergy development [99,100]. Since the former studies specified that infants were already presenting allergic predisposition, exposure would most likely contribute to the sensitization rather than priming. However, the presence of allergens and sIgE antibodies could contribute to prenatal allergic sensitization. Some studies have suggested that this exposure could prime the fetal immune response, similar to sensitization to food allergens [101]. The production of CD23b was reported in response to IL-4 and IL-13 from tissue samples harvested during the first and second pregnancy trimester. CD23b transcription declined during the third trimester and rose again after birth [92]. Similarly, gastrointestinal cells expressing CD23 on the surface were capable of binding IgE and IgE–antigen complexes. Via the CD23 receptor, these complexes could bypass the tight junctions of the epithelial barrier and the lamina propria where they bind to the FcεRI on mast cells and trigger degranulation [83,102] (Figure 2). In a similar fashion, maternal IgE was found to bind to the neonatal Fc receptor (FcRN) on fetal mast cells, inducing degranulation and priming the fetal immune response to induce class switching in B cells and allergic sensitization upon an allergen encounter [92,103].

Maternal exposure to different substances was also identified as a potential trigger of allergic predisposition in infants (Figure 5). The most frequently identified risk factor was exposure to cigarette smoke. Cigarette smoke in a mouse model of allergy was found to promote Th2 type cytokines in response to an antigen challenge [104]. The cigarette smoke-induced cytokine response could cause allergic diseases, since smoking during pregnancy was associated with reduced respiratory function in early infancy and recurrent wheezing in childhood [105,106]. Cigarette smoking was found to induce different methylation patterns, including in regions near genes involved in lung (*BMP4*—bone morphogenetic protein 4) and orofacial development (*BHMT2*—betaine-homocysteine S-methyltransferase 2) in the cord blood, which could further contribute to the development of allergic disorders such as asthma [82]. Additional factors found to drive the disequilibrium between Th1 and Th2 cells were chemicals found in personal care products, i.e., mono-*n*-butyl phthalate and methylparaben [107]. Monoethyl phthalate exposure during pregnancy was associated with decreased lung function in infants [107]. Exposure to perfluoroalkyl substances during pregnancy was associated with an increased risk of AD in childhood [108]. A recent study by Karmaus et al. investigated the effect of different metabolites, nutrients and toxins on various indicators of different allergy-related outcomes [109]. The authors found triacylglycerol, tryptamine and benzyl (2-ethylhexyl) phthalate as risk factors of increased FeNO, which were attributed to their role in the metabolism of inflammatory mediators, in the gut microbiome-driven immunity and by damaging the epithelial barrier, respectively [109]. Differences in fatty acid composition were found between allergic and non-allergic individuals, most notably lower levels of polyunsaturated fats in the cord blood of infants who developed AD or asthma in the first year of life [110].

The use of antibiotics during pregnancy was also found to increase the risk of asthma development in childhood [111]. Cord blood IgE was found to be increased in mothers which took metoprolol during pregnancy, suggesting that beta-adrenergic blockers could enhance the formation of IgE antibodies [112]. Also, the intake of progesterone during pregnancy was found to induce detectable IgE levels in the cord blood of 53% of newborns, compared to only 24% in the control group [113].

Other prenatal factors that have been associated with allergy development are birth season in autumn and/or winter, which could be due to the time of encounter and sensitization to seasonal allergens [90,114]. Male sex of the offspring also seemed to be associated with elevated levels of cord blood IgE [115]. The use of pesticides in the household did appear to increase total cord blood IgE levels in non-allergic mothers [90]. Social factors, including mental health disorders like anxiety and depression, have been associated with an increased risk of asthmatic outcomes in offspring [116,117].

## 3. Prenatal Protective Factors in the Development of Allergic Diseases

Since IgE antibodies and Th2 response were identified as the main drivers of allergic diseases, redirecting these responses towards IgG and Th1 is considered an important protective factor. The placental transfer of maternal IgG to the fetus was found to start during the second trimester of pregnancy and continue until birth [118,119]. The transferred IgG antibodies also included IgG against common inhalant allergens, like cat dander and pollen [120]. In a mouse model of allergy, the IgG levels were found to prevent the development of type I hypersensitivity reactions in offspring and to reduce the risk of sensitization towards these allergens even upon repeated exposures [121]. The effect was suggested to be induced by the neutralization of the antigen and the suppression of IgE binding [122,123].

Maternal immunizations increased the IgG1 and IgG2 antibody levels in mouse offspring [124]. Additionally, this immunization resulted in a stronger effect of reducing the number of B cells secreting IL-4 and IL-12 and of T cells secreting IL-4 and IFN-γ upon neonatal immunization [124]. The transfer of maternal IgG upon immunization with ovalbumin inhibited the IgE response in offspring and increased the production of T cells secreting regulatory cytokine IL-10 after exposing the offspring to the antigen [124]. Therefore, allergen-specific immunization during pregnancy to induce high levels of specific IgG antibodies has been proposed as a preventive strategy against allergy development [125]. Total IgE levels decrease with maternal age, as well as with the increasing number of live offspring, suggesting that the offspring of increasing birth order experience differences in the fetal immune system development, which could have a role in the prevention of allergy [89,115].

The Th2 type response at birth was found to be similar between children who developed allergy and normal children. During the first year of life, the normal Th2 response shifted towards the Th1 response upon exposure to different pathogens in normal children, whereas the Th2 response was consolidated in children who developed allergy [126]. The failure to mount a Th1 response appeared to be due to faulty production of IFN-γ [126]. A low level of expression of protein kinase C zeta (PKCζ) in cord blood T cells was also indicative of a faulty shift towards the Th1 response, having been proposed as a marker for the development of allergic diseases [76,127]. Additionally, early and even prenatal subacute microbial inflammation was considered to aid the proper development of immune response [102,128]. A low Th1 response rate to microbial antigens during pregnancy was found to stimulate a rapid shift towards the Th1 response in infants, a process known as the “farm effect” [129].

In several studies, maternal nutrition was found to influence the development of allergic diseases in offspring. Maternal diet was often investigated regarding the effect on different allergic diseases in offspring. Contrary to previous findings regarding peanut allergy, Bunyavanich et al. found that the consumption of allergenic foods during early pregnancy was beneficial in preventing allergic diseases at 7 years [130]. Peanut intake reduced peanut allergic reactions by 47%, while milk consumption reduced the risk of allergic rhinitis and asthma [130]. Similarly, wheat consumption during pregnancy reduced the odds of AD at 7 years [130]. The maternal diet index, including higher intake of vegetables and yogurt, was found to have a protective effect on the development of most allergic disorders, with the exception of food allergy [131]. This might be mediated in non-allergic mothers by IgG against these allergens, which are transferred via the cord blood. During pregnancy and through breast milk, maternal immunoglobulins are transferred to the offspring, and maternal allergen-specific IgG may protect the offspring from allergic sensitization [132,133]. A meta-analysis revealed that intake of vitamin D had a significant protective effect against infant wheeze and asthma, while intake of omega 3 fatty acids also tended to have a protective effect [134]. Similarly, insufficient intake of vitamin C and magnesium during pregnancy was associated with a risk of wheezing in infancy, whereas a lack of vitamin E was associated with a risk of AD [135]. Fish oil supplementation was also found to increase the expression of PKCζ in cord blood T cells via histone modifications in the promoter region, which further shifted the T-cell response to the production of Th1 cytokines [136]. The maternal intake of n-3 polyunsaturated fatty acids was associated with a decrease in rhino-conjunctivitis and asthma when the infants were 3 years old [137]. Different nutrients and metabolites in maternal and cord blood have been associated with a protective role in lung function against asthma and elevated IgE levels of offspring [109]. The protective role in lung function has been attributed to the substances with antioxidant and anti-inflammatory properties in the gut microbiome and those playing a role in the maintenance of the lung epithelium. The exposure to a sulfate conjugate of polyphenol from maternal and cord serum resulted in increased FVC and FEV1 at 26 years, attributed to its role as an antioxidant with anti-inflammatory properties in fruits and vegetables. Similarly, 7-amino-4-hydroxy-2-naphthalenesulfonic acid was associated with a protective effect on FEF_25–75%_ of female offspring at 6 years of age, possibly playing a role in the antioxidant properties of gut microbiome metabolism. Additionally, polyunsaturated phosphatidylglycerol detected in maternal and cord serum was related to increased FEV1 and FVC, suggesting a lung-protective effect by integration in the phospholipids found in lung surfactants. Hypoxanthine had a protective effect for FEV1 across two generations of male offspring due to its role in nucleotide metabolism. A bile acid metabolite was associated with decreased IgE levels, attributed to the function of the gut microbiome, which could drive T-cell differentiation towards tolerance [109].

## 4. Conclusions

Allergic diseases are health conditions of increasing prevalence worldwide. They are characterized by an exaggerated response towards normally harmless environmental stimuli. Allergic diseases start developing with skin symptoms during early childhood and progress towards respiratory symptoms in adolescence and adulthood. Mutations and the regulation of genes involved in the maintenance of the epithelial barrier function and T-cell differentiation are among the genetic and epigenetic factors driving allergic diseases. Environmental factors also play a role in dysfunction of the epithelial barrier, T-cell differentiation and allergen sensitization. Disease development in predisposed children is further influenced by prenatal risk factors, including maternal and cord blood IgE, as well as maternal exposure to cigarette smoke and pesticides. Protective factors include maternal IgG, the intake of vitamin D and omega 3 fatty acids, and metabolites involved in the immune regulation of the gut microbiome. Sub-pathogenic microbial exposure also seems to help shift the immune response towards tolerance. Contradictory evidence can be found regarding the consumption of allergenic foods; therefore, dietary recommendations regarding allergen avoidance are lacking. Finally, genetic and environmental factors play a role in the development of allergic diseases by inducing dysfunctions in epithelial barrier integrity and influencing T-cell differentiation.

## Figures and Tables

**Table 1:** The three major types of cell-mediated immunity (Type IVa-c) according to Jutel et al. and Annunziato et al. [8,10].

	Type I Response (IVa)	Type II Response (IVb)	Type III Response (IVc)
**Role**	Intracellular pathogen (viruses, *Mycobacterium tuberculosis*)	Helminths, poisons, venoms	Extracellular pathogens (bacteria, fungi)
**Key components**	Type 1 CD4+ T cells (Th1) Type 1 innate lymphoid cells (ILC1) Natural Killer cells (NK) Natural Killer T cells (NK-T) Type 1 CD8+ cytotoxic lymphocytes (Tc1)	Type 2 CD4+ T cells (Th2) Type 2 innate lymphoid cells (ILC2) Type 2 CD8+ cytotoxic lymphocytes (Tc2)	Type 3 CD4+ T cells (Th17) Type 3 innate lymphoid cells (ILC3) Type 3 CD8+ cytotoxic lymphocytes (Tc17)
**Mediators**	Interferon-gamma (IFN-γ), Tumour Necrosis Factor (TNF), Interleukin-12 (IL-12)	IL-4, IL-5, IL-9, IL-13, (IL21)	IL-8, IL-17, IL-22, TNF, Granulocyte-macrophage colony-stimulating factor (GM-CSF)
**Antibodies**	IgG1, IgG2, IgG3	IgE	
**Effector cells**	Macrophages, neutrophils	Eosinophils, mast cells, basophils	Mononuclear phagocytes, neutrophils

## Data Availability

Not applicable.
